# Phytochemical, Antibacterial and Antioxidant Activity Evaluation of *Rhodiola crenulata*

**DOI:** 10.3390/molecules25163664

**Published:** 2020-08-12

**Authors:** Lingyun Zhong, Lianxin Peng, Jia Fu, Liang Zou, Gang Zhao, Jianglin Zhao

**Affiliations:** 1 College of Medicine, Chengdu University, Chengdu 610106, Sichuan, China; zhongly@cdu.edu.cn (L.Z.); j_f_cdu@163.com (J.F.); 2 Key Laboratory of Coarse Cereal Processing, Ministry of Agriculture and Rural Affairs, Chengdu 610106, Sichuan, China; penglianxin@cdu.edu.cn (L.P.); bsmpmcereal@163.com (L.Z.); ccpczhaogang@163.com (G.Z.)

**Keywords:** *Rhodiola crenulata*, essential oil, phenols, antibacterial, antioxidant

## Abstract

The chemical components, as well as the antibacterial and antioxidant activities of the essential oil (EO) and crude extracts prepared from *Rhodiola crenulata* were investigated. The essential oil was separated by hydrodistillation, and gas chromatography-mass spectrometry (GC-MS) was used to identify its constituents. A total of twenty-seven compounds was identified from the EO, and its major components were 1-octanol (42.217%), geraniol (19.914%), and 6-methyl-5-hepten-2-ol (13.151%). Solvent extraction and fractionation were applied for preparing the ethanol extract (crude extract, CE), petroleum ether extract (PE), ethyl acetate extract (EE), *n*-butanol extract (BE), and water extract (WE). The CE, EE and BE were abundant in phenols and flavonoids, and EE had the highest total phenol and total flavonoid contents. Gallic acid, ethyl gallate, rosavin and herbacetin were identified in the EE. The antibacterial activity results showed that the EO exhibited moderate inhibitory activity to the typical clinic bacteria, and EE exhibited the strongest antibacterial activity among the five extracts. For the compounds, ethyl gallate showed the strongest inhibitory activity to the test bacteria, and its minimum inhibitory concentration (MIC) value and minimum bactericidal concentration (MBC) value for all the tested bacteria was 0.24 mg/mL and 0.48 mg/mL, respectively. The results of antioxidant activity showed that both CE and EE exhibited strong antioxidant activities in the DPPH radical scavenging and Fe^2+^ reducing power tests, however, EO showed relatively weaker antioxidant ability. Ethyl gallate and rosavin exhibited excellent activity in the DPPH radical scavenging assay, and their IC_50_ value was 5.3 µg/mL and 5.9 µg/mL, respectively. Rosavin showed better reduction power activity than the other three compounds. These results could provide more evidence for the traditional use of *R. crenulata*, and would be helpful for improving its application further.

## 1. Introduction

*Rhodiola* L., which belongs to the Crassulaceae, includes plenty of valuable medicinal alpine plant resources. There are about 200 species of *Rhodiola* around the world, and the *Rhodiola* resources are mainly distributed in the Himalayan region, the west to northern part of Asia and northern America [1,2]. *Rhodiola* has appeared in the materia medica of numerous countries, and its extracts have been widely used throughout the United States, Europe and Asia [3,4]. Many high-value or well known herbal medicinal products of *Rhodiola* are commercially available, such as *Rhodiola* capsule, *Rhodiola* tablets and *Rhodiola* oral liquid. These *Rhodiola* preparations are mainly made from the rhizomes of *Rhodiola*, and they usually have significant effects on relieving stress-induced fatigue, exhaustion, anxiety, etc. [5,6,7]. In the traditional Chinese medicine, *Rhodiola* sp. was used for treating diarrhea, scald, neural paralysis and erysipelas. Gradually, the hemostatic agents, or endermic liniments for burns and contusions which contained ingredients of *Rhodiola* were developed [8,9,10].

*R. crenulata* is one of the important species in the genus of *Rhodiola*, and its rhizome has been successfully recorded by the Chinese Pharmacopoeia [11,12]. In China, *R. crenulata* is considered as the only official species, and it has been widely recognized as the high-quality raw material for making various products, including medicine, health-preserving food and cosmetics [13,14]. Years of pharmacological studies have revealed that, the extracts of *R. crenulata* can improve memory, adjust immune, anti-hypoxia, anti-inflammatory, anti-apoptotic, anti-tumor, resist fatigue, and have the function of dual-directional regulation in accommodating the central nervous system and endocrine system [15,16,17,18,19]. To date, over 100 compounds, such as salidroside, arbutin, gallic acid, ethyl gallate, tyrolsol, kaempferol, herbacetin, rhodiolinin and rosavin, have been successfully separated and characterized from this plant [20,21,22,23,24]. These chemical constituents can be generally grouped into the categories of organic acids, phenyethanoids, phenols, polyphenols, terpenoids, lignans, flavonoids, flavonoids glycosides, cyanogens, and cyanophoric glycosides. Many components obtained from *R. crenulata* have been screened for their bioactivities, and some of them had been reported to exhibit various pharmacological activities. It was found that the Kaempferol, salidroside, arbutin and rhodiolinin identified from *R. crenulata* had excellent antioxidant capacity and could significantly improve the irradiation-induced cell apoptosis [25]. The polyphenol compounds epicatechin-(4 beta, 8)-epicatechingallate (B2-3′-*O*-gallate), epicatechin gallate (ECG), and epicatechin (EC) isolated from *R. crenulata* showed strong inhibitory activity to the maltase and sucrase [26]. In addition, (-)-Epicatechin gallate ((-)-ECG), 1,2,3,4,6-*O*-pentagalloylglucose (PGG), rhodionin, herbacetin and rhodiosin exhibited potent inhibitory effects on acetylcholinesterase (AChE) with the median effective inhibitory concentration (IC_50_) value ranging from 2.43 to 57.50 mu g/mL [27].

The biological functions of the rhizomes extracts of *R. crenulata* have been studied for many years, and their outstanding pharmacological effects have also been gradually revealed. However, the chemical basis, as well as the various bioactivities of this well known medicinal plant still need to be further elucidated. In addition, the essential oil (EO) extracted from *R. crenulata* has also aroused the interests of many researchers. It was reported that about 60 volatile components have been identified from the essential oil of *R. crenulata* by GC-MS technique, and geraniol and octanol were usually the major compounds identified from the essential oil [28,29]. Nevertheless, the antibacterial and antioxidant activity of the essential oil of *R. crenulata* has never been reported up to now. Moreover, it is interesting and meaningful to find out whether the essential oil would play a role in its pharmacological functions.

**Figure 1:**
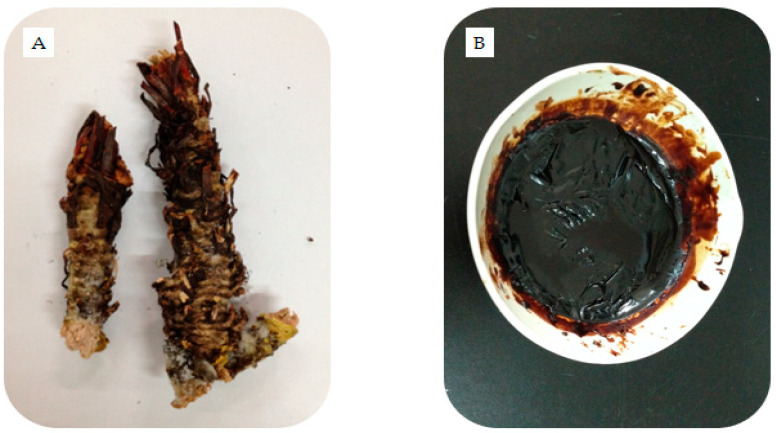
The rhizomes (**A**) and the ethanol extract (**B**) prepared from *R. crenulata.*

In this study, the healthy rhizomes of *R. crenulata* were collected, and the essential oil and crude extracts of *R. crenulata* were prepared firstly (Figure 1). Then, the chemical compositions of the essential oil were preliminary determined, and the key components of the most active extract (EE) were analyzed further by the high-performance liquid chromatography (HPLC) method. Moreover, the crude extracts with different polarity, the essential oil, and the compounds identified from the extract were screened for their antibacterial and antioxidant activities, which would help to reveal the material basis of the traditional use of *R. crenulata* in the Chinese Pharmacopoeia.

## 2. Results and Discussion

### 2.1. Chemical Composition of the Essential Oil

The light yellow essential oil (EO) with fragrant flavor was successfully obtained from the rhizomes of *R. crenulata* by steam distillation extraction. The yield (*v*/*w*) of EO was determined as 0.27%. Results of GC–MS analysis revealed the EO was abundant in alcohols, monoterpenes, ketones, aldehydes, esters, alkanes and acids (listed in Table 1). Notably, the alcohols and monoterpenes accounted nearly for over 95% of the total oil. The major components of EO were 1-octanol (42.217%), geraniol (19.914%), 6-methyl-5-hepten-2-ol (13.151%), prenol (4.21%), linalool (3.89%), 1-decanol (3.35%), 3-methyl-1-buten-3-ol (2.92%), and 1-hexanol (2.42%). Generally, many of the volatile constituents identified in this study had been reported by other articles [29,30]. However, the contents of each compound might be diverse in different researches. In our research, the amount of 1-octanol was the highest, followed by the geraniol. However, in Lei’s research, geraniol was the most abundant with the concentration of 53.32%, and 1-octanol listed the second with an amount of 13.39% [30]. There remained differences in the constitutions of essential oil from *R. crenulata* which were collected at different locations, and it might infer that the chemical constitution and amount of *R. crenulata* essential oil was closely related with its habitat [31]. Of course, several other factors might also affect its chemical composition and content, such as harvesting conditions, the level of growth and maturation, storage and handling conditions, etc.

Comparing with *R. rose*, which is available as another important raw material, mainly occurring in European markets, the same volatile components of *R. crenulata* were found, namely octanol, geraniol, linalool, hexanol, myrtenol and hexanal. However, it was reported that the major components in the essential oil of *R. rose* were tetrahydronootkatone (18.31%), geraniol (16.46%), trans-pinocarvone (15.71%), octanol (11.43%) and myrtenol (11.29%) [32,33], which were obviously different from those of *R. crenulata*.

### 2.2. Chemical Compositions of the Crude Extracts

The ethanol extract (crude extract, CE) was fractionated by organic solvents, and afforded four extracts, which were petroleum ether extract (PE, 5.21 g), ethyl acetate extract (EE, 12.47 g), *n*-butanol extract (BE, 43.20 g), and water extract (WE, 12.93 g). The yields of these five crude extracts are represented in Table 2. The total phenolics content of the five extracts of *R. crenulata* was measured in gallic acid equivalent (GAE), and the total flavonoids was calculated in milligrams of rutin equivalent (RE) per gram of extract. As shown in Table 2, the total phenols’ content of five extracts ranged from 12.0 to 172.0 mg GAE/g extract, and the flavonoids content was from 0 to 163.0 mg SRE/g extract. Generally, the CE, EE and BE were all rich in phenols and flavonoids. Especially, of these five extracts, EE contained the highest quantities of total phenols and flavonoids, with the amount of 171.89 mg GAE/g and 162.04 mg RE/g, respectively. The total phenols content of CE was about 134.91 mg GAE/g, which was lower than the EE, but higher than that of the BE, WE and PE. Moreover, the total flavonoids content of CE and BE was nearly the same, with no significant difference. Nevertheless, the flavonoids were not detected in the crude extracts of PE and WE.

**Figure 2:**
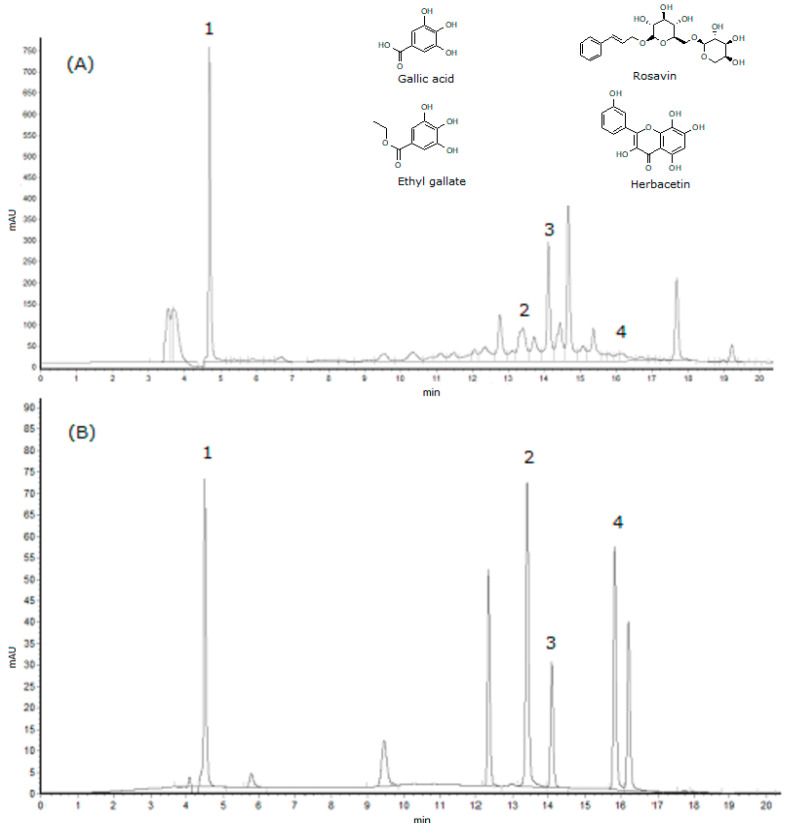
The HPLC chromatograms of ethyl acetate extract (**A**) and the standard references (**B**) at 275 nm; 1—gallic acid; 2—ethyl gallate; 3—rosavin, and 4—herbacetin.

In our previous preliminary investigation, we found that the EE exhibited good biological activity among the five crude extracts. It is very meaningful to find out which kinds of components could play a key role in the bioactivity of EE. Therefore, the EE was selected to be subjected to the HPLC analysis for the purpose of determining the relative active compounds. By comparing the retention times with the standards, a total of four compounds in the EE of *R. crenulata* were successfully identified, which were gallic acid (1), ethyl gallate (2), rosavin (3), and herbacetin (4) (listed in Figure 2). The content of gallic acid, ethyl gallage and rosavin in EE were determined as 4.13 ± 0.23 mg/g, 0.41 ± 0.06 mg/g, and 2.85 ± 0.17 mg/g, respectively.

### 2.3. Antibacterial Activity

#### 2.3.1. Antibacterial Activity of the Essential Oil and the Crude Extracts

Antibacterial test of the essential oil and five crude extracts were conducted by micro-broth-dilution-colorimetric assay. Three Gram-negative bacteria (*Shigella dysenteriae*, *Escherichia coli*, and *Salmonella typhimurium*), which usually cause severe intestinal infections, and two Gram-positive bacteria (*Staphylococcus aureus* and *S. albus*), which can cause pyogenic infection, were chosen as the test bacteria. As presented in Table 3, the EO of *R. crenulata* showed moderate antibacterial activity in this study. It could inhibit the visible growth of *S. dysenteriae* and *E. coli* at the concentration of 5.0 mg/mL. Accordingly, their minimum bactericidal concentration (MBC) value was determined to be 10.0 mg/mL. However, the EO was not active to the two Gram-positive cocci, and their minimum inhibitory concentration (MIC) value should be more than 5.0 mg/mL.

For the antibacterial activity of the five crude extracts of *R. crenulata,* they showed different antibacterial capacity. Generally, CE and EE displayed good antibacterial activity. Notably, EE exhibited the strongest antibacterial activity among the five extracts, which significantly inhibited the growth of all five bacteria strains. In this test, it was also observed that the Gram-positive bacteria seemed more sensitive than the Gram-negative bacteria to the extracts. Overall, EE inhibited the visible growth of *S. aureus*, *S. albus* and *S. dysenteriae* at the concentration of 1.25 mg/mL. In addition, the MIC value of EE to *E. coli* and *S. typhimurium* was 2.5 mg/mL. And the MBC values of EE to *S. aureus*, *S. albus, S. dysenteriae* and *E. coli* ranged from 2.5 to 10.0 mg/mL. However, the *S. typhimurium* was shown to be less sensitive to EE, and its MBC value was not detected at the maximum concentration of 10.0 mg/mL. Additionally, CE exhibited stronger antibacterial activity than BE, and the MIC value of CE to all the tested bacteria was 2.5 mg/mL, except *S. dysenteriae*. The MIC values of BE to *S. dysenteriae*, *E. coli*, *S. aureus* and *S. albus* were 5.0 mg/mL, 2.5 mg/mL, 2.5 mg/mL, and 2.5 mg/mL, respectively. However, it was not active to *S. typhimurium*. The PE and WE did not show any antibacterial activity to all the test bacteria at the maximum concentration of 10.0 mg/mL. In all, the inhibitory activity of the five crude extracts against the tested bacteria was in the order of EE > CE > BE > PE ≈ WE, which was the same as the order of total phenol content. This implies that the total phenol content of extracts might closely correlate with the antibacterial activity. From these results, it could be inferred that the antibacterial properties of the crude extracts from *R. crenulata* might confirm its popular use of treating diarrhea and scald [34].

#### 2.3.2. Antibacterial Activity of the Compounds

As shown in Table 4, gallic acid, ethyl gallate, and herbacetin showed excellent inhibitory activity against the test bacteria, except rosavin. Of them, ethyl gallate exhibited the strongest antibacterial activity, and its MIC value to the five typical test bacteria was 0.24 mg/mL, and its MBC value was determined as 0.48 mg/mL. Herbacetin showed better inhibitory activity to *S. typhimurium* and *S. dysenteriae* than gallic acid, and its MIC values against the two bacteria strains was 0.24 mg/mL. It could be concluded that ethyl gallate, gallic acid and herbacetin were three important components which could inhibit the growth of the five clinical pathogenic bacteria, and they might contribute to the antibacterial activity of EE. These results would provide more clues to reveal the material basis of *R. crenulata* in treating diarrhea and scald, which would also help to understand the traditional use in Chinese traditional medicine [35]. Furthermore, the antibacterial mechanism of the crude extracts and the identified compounds need to be elucidated more clearly, and an in vivo antibacterial activity experiment is required to be conducted in further study.

### 2.4. Antioxidant Activity

For investigating the antioxidant activity of the essential oil, crude extracts, and compounds identified from *R. crenulata*, the typical DPPH radical scavenging and Fe^2+^ reducing power assays were employed.

#### 2.4.1. Antioxidant Activity the Essential Oil and Extracts

Results of DPPH radical scavenging activity of the crude extracts were shown in Table 5. All the five extracts exhibited a DPPH radical scavenging ability in a dose-dependent manner. In general, CE exhibited the strongest radical scavenging capacity among the five extracts, and its IC_50_ value was determined as 20.3 µg/mL. This was followed by the EE and BE, as they all exhibited excellent radical scavenging ability with IC_50_ values of 24.1 µg/mL and 36.1 µg/mL, respectively. However, the scavenging ability of the DPPH radical by WE (IC_50_ = 130.5 µg/mL) and PE (IC_50_ = 321.8 µg/mL) appeared to be relatively weaker. Totally, the DPPH radical scavenging activity was in the order of CE > EE > BE > WE> PE > EO.

**Figure 3:**
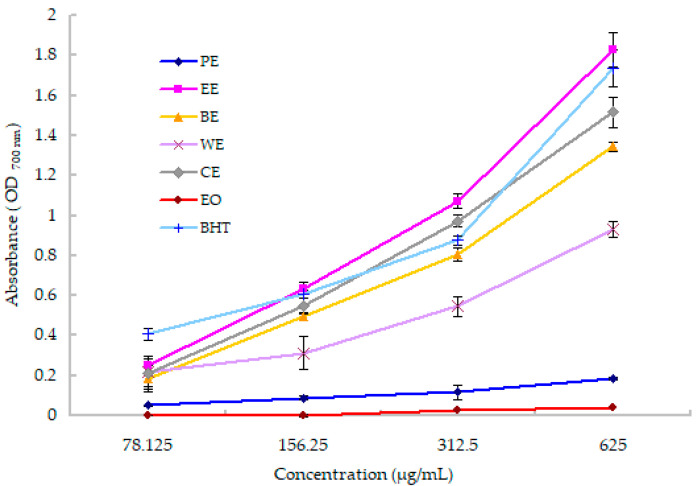
Reducing powers of the essential oil and five crude extracts of *R. crenulata.* The CE, PE, EE, BE and EO represent the crude extract, petroleum ether extract, ethyl acetate extract, *n*-butanol extract and essential oil, respectively. BHT is the butylated hydroxytoluene.

For many cases, antioxidant substances may have the reducing power ability [36]. In the reducing power assay, the presence of reductants can cause the reduction of Fe^3+^/ferricyanide complex to the ferrous form, and form Perl’s Prussian blue with ferric chloride [37]. The Prussian blue can be measured at 700 nm, and the absorbance may serve as an indicator of antioxidant capacity. The higher absorbance indicated stronger potential antioxidant activity. Figure 3 illustrated the reducing power activity of the essential oil and five crude extracts. At different concentrations (78.125–625 µg/mL), these test samples exhibited different reducing abilities. In all, the reduction power was gradually increased with the increasing concentrations. Among the extracts and EO, EE displayed the best reducing activity, followed by the CE, BE and WE. However, the PE and EO displayed weak or no reducing power in this test. Generally, the five extracts and EO showed different activity in the two different antioxidant models, and both CE and EE exhibited strong antioxidant activities in the DPPH radical scavenging and Fe^2+^ reducing power tests. The reducing power ability was in the order of EE > CE > BE > WE > PE > EO, which was similar to that of DPPH scavenging activity, except for EE and CE.

#### 2.4.2. Antioxidant Activity of the Compounds

The DPPH radical scavenging activity results from four identified compounds, which are presented in Table 6. Of them, ethyl gallate and rosavin displayed excellent radical scavenging activity, with the IC_50_ value of 5.33 µg/mL and 5.86 µg/mL, respectively, which were stronger than the positive control butylated hydroxytoluene (BHT) (IC_50_ = 17.96 µg/mL) in this study. Gallic acid exhibited similar ability with the positive control, and its IC_50_ value was calculated as 18.03 µg/mL. Comparing with the crude extracts of CE and EE, herbacetin showed similar DPPH radical scavenging activity, but it was weaker than that of the other three identified compounds. On the whole, the DPPH radical scavenging activity was in the order of ethyl gallate ≈ rosavin > gallic acid ≈ BHT > herbacetin.

**Figure 4:**
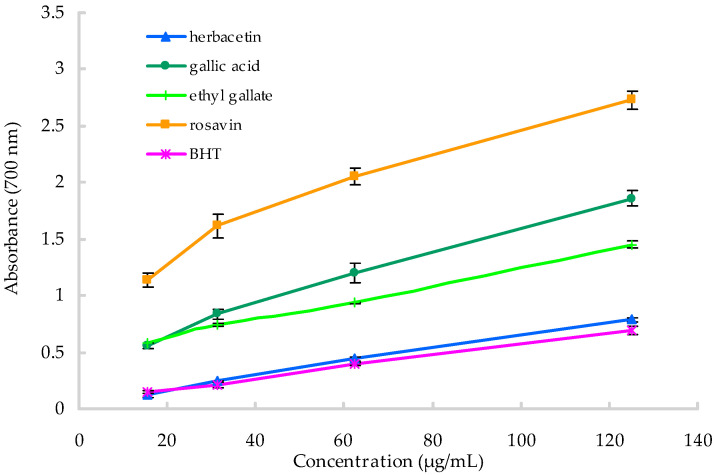
The reducing power of the four compounds of *R. crenulata.*

Figure 4 illustrates the reducing power activity of the four identified compounds of *R. crenulata*. Generally, the four compounds exhibited good reducing ability, and their antioxidant activity was concentration-dependent. Moreover, their reduction power was in the order of rosavin > gallic acid > ethyl gallate > herbacetin > BHT. Furthermore, the antioxidant activity of the four identified compounds was higher than that of the essential oil or crude extracts. Excellent antioxidant activity of the extracts and compounds from *R. crenulata* might be closely relate to its pharmacological functions, such as anti-aging effect [38].

## 3. Experimental

### 3.1. Plant Material

The fresh rhizomes of *R. crenulata* were collected from the Hongyuan county of Aba, Sichuan Province of China in August 2016, and a voucher specimen (CCPC-12) of this plant was deposited at the Coarse Cereal Research & Development Center of Chengdu University. Dr. Peng Lianxin of Chengdu University authenticated the taxonomic identity of the plant material.

### 3.2. Preparation of the Essential Oil

The fresh rhizomes of *R. crenulata* (300.0 g, fresh weight) were submitted to hydrodistillation in a Clevenger-type apparatus at 100 °C for 5.5–6 h. The essential oil (EO) was successfully separated according to the method described by Zhao [39], and stored at 4 °C in dark.

### 3.3. Preparation of the Crude Extractss

The air-dried and powdered rhizomes of *R. crenulata* (1400.0 g, dry weight) were extracted with 95% ethanol under reflux for three times, and 3 h for each time. The combined ethanol solution was concentrated dryness to obtain the crude extract (CE) (101.0 g). A part of the crude extract (80.0 g) was further suspended in hot water, and extracted with petroleum ether, ethyl acetate and n-butanol in turn. Subsequently, these solutions were concentrated in vacuum, respectively, to obtain the petroleum ether extract (PE), ethyl acetate extract (EE) and *n*-butanol extract (BE) and water extract (WE).

### 3.4. Chemical Analysis

#### 3.4.1. Chemical Analysis of the Essential Oil

The chemical compositions of the essential oil of *R. crenulata* rhizomes were analyzed by GC-MS techniques. A 6890N Network GC system (Agilent, CA, USA) equipped with a HP-5 MS capillary column (30m × 0.25mm, 0.25μm film thickness) was used. Carrier gas helium was run at a flow rate of 1.0 mL/min, and the temperature program run from 50 °C (2 min), increased 10 °C/min up to 100 °C, and 2 min at 180 °C, increased 20 °C/min up to 250 °C. Moreover, the GC-MS experiment was run for one time. The chemical constituents of the essential oil were identified by comparing their mass spectra to those in the NIST (National Institute of Standards and Technology) library and those from the literature.

#### 3.4.2. Determination of the Total Phenols and Total Flavonoids of the Crude Extracts

The quantification of the total phenols of the extracts was performed according to the Folin-Ciocalteu method described by Bujor [40] with some modifications. Approximately 0.1 mL of each extract solution (5000 μg/mL) or gallic acid (62.5–1000 μg/mL) was mixed with 7.9 mL of distilled water and 0.5 mL Folin-Ciocalteu’s reagent. Then, 1.5 mL of 20% sodium carbonate was added to the mixture mentioned above at 30 °C for 1 h. The absorbance was read at 750 nm by a spectrophotometer. The total phenol content was expressed as the milligram of gallic acid equivalent (GAE) per gram of extract.

The content of the total flavonoids was determined by the colorimetric method of Sharma [41] and Qin [42] with small modifications. About 0.5 mL of each extract (1875 μg/mL) or rutin (28–240 μg/mL) was mixed with 1.5 mL of 10% aluminum chloride, 3 mL of 1M potassium acetate and 5 mL of 75% ethanol. The reaction mixture was maintained for 40 min at room temperature, and the absorbance was measured against a blank at 415 nm. Rutin was used for establishing the standard calibration curve, and the total flavonoids content was calculated in milligrams of rutin equivalent (RE) per gram of extract.

#### 3.4.3. Chemical Analysis of the Ether Acetate Extract by HPLC

The ether acetate extract with notable bioactivity was subjected to the HPLC analysis on a LC-10ATvp system. Sample analysis was performed using a C_18_ column (4.6 mm × 250 mm, 5 μm, Phenomenex, Torrance, CA, USA), and an SPD-M10Avp diode-array detector (Shimadzu, Kyoto, Japan) which recorded at 275 nm. The mobile phase was A (water with 2.5% acetic acid) and B (acetonitrile 100%). The HPLC procedure was as follows: 0–2 min, 15% B; 2–10 min, 50% B; 10–30 min, 90% B; 30–40 min, 100% B. The flow rate was 1.0 mL/min at 35 °C, and the infection volume was 20 μL. Seven pure phenolic compounds, including gallic acid, ethyl gallate, rosavin, rutin, quercetin, herbacetin and kaempferol, were used as the reference standards for the identification of the active components in EE. All the standards were purchased from Sichuan Weikeqi Biological Technology Co., Ltd. The quantification of the flavonoids was obtained by using an external standard method [43].

### 3.5. Antibacterial Activity Test

A modified micro-broth-dilution-colorimetric assay was applied to detect the antibacterial activity [44]. Test microorganisms included two Gram-positive (*Staphylococcus aureus* ATCC 6538 and *S. albus* CICC 10897), and three Gram-negative (*Shigella dysenteriae* CMCC 51105, *Escherichia coli* ATCC 25922, *Salmonella typhimurium* CMCC 50115) bacteria. ATCC referes to American type culture collection, CICC is the abbreviation for China center of industrial culture collection, and CMCC referes to China national center for medical culture collections. Test bacteria were grown in liquid Luria-Bertani (LB) medium (yeast extract 5.0 g/L, peptone 10.0 g/L, NaCl 5.0 g/L, pH 7.2) for 24 h at 37 ºC, and the diluted bacterial suspension (1 × 10^6^ CFU/mL) was ready for detection. The extracts and four phenolic compounds were dissolved and diluted with 60% methanol (40% acetone for essential oil) to obtain appropriate concentrations in the range of 0.1 mg/mL to 100 mg/mL, respectively. Then, the test sample solutions (10 μL) and prepared bacterial suspension (90 μL) were added into each well of the 96-well microplate. Each well of the negative control (CK^−^) contained 90 μL of the inoculum and 10 μL of 60% methanol or 40% acetone. Streptomycin sulfate was used as the positive control (CK^+^). The 96-well plates were agitated to mix the contents of the wells using a plate shaker, and then incubated in the dark at 37 °C for 24–48 h. The minimum inhibitory concentration (MIC) value was defined as the lowest sample concentration that inhibited visible growth. To determine the minimum bactericidal concentration (MBC), 10 μL of suspension liquid was removed from the well and inoculated on the LB media, and incubated at 37 °C overnight. The MBC value was determined as the highest dilution at which no growth occurred on the media. All tests were performed in triplicate.

### 3.6. Antioxidant Activity

#### 3.6.1. DPPH Radical Scavenging Assay

The DPPH (1,1-diphenyl-2-picrylhydrazyl) radical scavenging assay was conducted in a 96-microplate according to the method of Wang [45]. Briefly, 80 µL of DPPH solution (0.2 mg/mL) and 20 µL of sample solutions of different concentrations were added to each well of the microplate and mixed. Then, the plates were incubated at 37 °C for 30 min in the dark. The absorbance at 515 nm was measured by a microplate spectrophotometer. The inhibition rate of free radicals was determined as (ODc-ODs)/ODc, where ODc is the absorbance of the negative control, and ODs is the absorbance of the test sample. Butylated hydroxytoluene (BHT) was used as the positive control. The median effective inhibitory concentration (IC_50_) value of each extract and identified compound were calculated, respectively, using a linear relation between the test sample concentration and the probability of the percentage of DPPH inhibition. All tests were run in triplicate.

#### 3.6.2. Reducing Power Assay

The measurement of reducing power was performed using the method of Demirtas [46] and Fu [47] with slight modifications. Briefly, 25 µL of different concentrations (10–1000 µg/mL) of extracts or compounds were mixed with 25 µL of 0.2 M sodium phosphate buffer (pH 6.6) and 25 µL of 1% potassium ferricyanide, and the mixture was incubated at 50 °C for 20 min. Then, 25 µL of 10% trichloroacetic acid and 5 µL of 1% ferric chloride were added to the mixture. The absorbance was measured at 700 nm. BHT was used as the positive control, and ethanol was the negative control. All the tests were carried out in triplicate.

### 3.7. Statistical Analysis

All tests were performed in triplicate, and the results were expressed by their mean values and standard deviations (SD). The experimental data were submitted to the analysis of their variance to determine significant differences by PROC ANOVA of SAS version 9.2 (SAS Institute Inc., Cary, NC, USA). The term significant difference was based on *p* ≤ 0.05.

## 4. Conclusions

The essential oil of fresh rhizomes of *R. crenulata* was rich in alcohols and monoterpenes, and its major components were 1-octanol (42.217%), geraniol (19.914%) and 6-methyl-5-hepten-2-ol (13.151%). The results showed that ethanol extract (crude extract, CE), ethyl acetate extract (EE), and *n*-butanol extract (BE) were abundant in phenolic acids and flavonoids. Both CE and EE exhibited strong activities in the antibacterial and antioxidant assays. Four compounds were successfully identified from the ethyl acetate extract. Of them, rosavin displayed excellent antioxidant activity both in the DPPH radical scavenging assay and the Fe^2+^ reducing power assay. Ethyl gallate, gallic acid and herbacetin could strongly inhibit the growth of important clinic pathogenic bacteria, and they play an important role in the antibacterial activity of the extract. This research firstly reported the antibacterial and antioxidant activities of the essential oil of *R. crenulata.* Additionally, the antibacterial and antioxidant activities of the five crude extracts from *R. crenulata* and the bioactive constituents in the extract were also investigated. These results may provide more evidence for the traditional use of the rhizomes of *R. crenulata* in treating diarrhea and scald, and would be helpful for improving its application further.

## Figures and Tables

**Table 1:** Chemical composition of the essential oil of *R. crenulata.*

NO.	Compound	Molecular Formula	Retention Time (Rt)	Relative Amount (%)
1	3-Methyl-1-buten-3-ol	C_5_H_10_O	2.23	2.921
2	Prenol	C_5_H_10_O	4.31	4.211
3	Hexanal	C_6_H_12_O	4.67	0.042
4	1-Hexanol	C_6_H_14_O	6.55	2.423
5	6-Methyl-5-hepten-2-one	C_8_H_14_O	9.98	0.138
6	6-Methyl-5-hepten-2-ol	C_8_H_16_O	10.52	13.151
7	2-Phenylethanal	C_8_H_8_O	11.79	0.105
8	1-Octanol	C_8_H_18_O	13.4	42.217
9	l-Linalool	C_10_H_18_O	13.88	3.886
10	Linalyl propionate	C_13_H_22_O_2_	16.37	0.334
11	Myrtenol	C_10_H_16_O	16.57	0.889
12	*n*-Octyl acetate	C_10_H_20_O_2_	16.83	0.274
13	(*R*)-(+)-*beta*-Citronellol	C_10_H_20_O	17.39	1.416
14	Geraniol	C_10_H_18_O	18.51	19.914
15	1-Decanol	C_10_H_22_O	18.77	3.35
16	*p*-Cymen-7-ol	C_10_H_14_O	19.26	0.174
17	Theaspirane B	C_13_H_22_O	19.39	0.074
18	Perillol	C_10_H_16_O	19.47	0.155
19	*trans, trans*-2,4-Nonadienal	C_9_H_14_O	19.82	0.23
20	*p*-Mentha-1,4-dien-7-ol	C_10_H_16_O	20.22	0.054
21	2,6,6-Trimethyl-1- cyclohexene-1-ethanol	C_11_H_20_O	20.33	0.061
22	Eugenol	C10H_12_O_2_	20.94	0.08
23	Geranyl acetate	C_12_H_20_O_2_	21.51	0.222
24	Dihydro-*beta*-ionone	C_13_H_22_O	22.94	0.056
25	Dihydro-*beta*-ionol	C_13_H_24_O	23.23	1.407
26	*alpha*-Cedrol	C_15_H_26_O	27.05	0.067
27	Hexadecanoic acid	C_16_H_32_O_2_	32.27	0.27

**Table 2:** Total flavonoids and phenolics contents of the five crude extracts of *R. crenulata.*

Extracts	Yields (%)	Total Phenolics (mg/g)	Total Flavonoids (mg/g)
PE	6.51	12.30 ± 0.09 ^e^	Nd
EE	15.59	171.89 ± 2.05 ^a^	162.04 ± 4.21 ^a^
BE	53.95	129.07 ± 0.24 ^c^	92.98 ± 0.85 ^b^
WE	16.16	34.04 ± 3.19 ^d^	Nd
CE	7.20	134.91 ± 1.69 ^b^	91.47 ± 1.03 ^b^

The CE, PE, EE, and BE represent the crude extract, petroleum ether extract, ethyl acetate extract, and *n*-butanol extract of *R. crenulata*, respectively. Values represent the mean ± standard deviation (*n* = 3). Different letters (i.e., a–e) indicate significant differences among the treatment at *p* = 0.05 level, Nd, not detected.

**Table 3:** The MIC and MBC values of the essential oil and five crude extracts of *R. crenulata* against pathogenic bacteria.

Test Sample	MIC/MBC (mg/mL)				
	*S. dysenteriae*	*S. typhimurium*	*E. coli*	*S. aureus*	*S. albus*
PE	>5.0/>10.0	>5.0/>10.0	>5.0/>10.0	>5.0/>10.0	>5.0/>10.0
EE	1.25/2.5	2.5/>5.0	2.5/10.0	1.25/2.5	1.25/2.5
BE	5.0/>10.0	>5.0/>10.0	2.5/>10.0	2.5/>10.0	2.5/>10.0
WE	>5.0/>10.0	>5.0/>10.0	>5.0/>10.0	>5.0/>10.0	>5.0/>10.0
CE	2.5/10.0	5.0/>10.0	2.5/>10.0	2.5/10.0	2.5/10.0
EO	5.0/10.0	>5.0/>10.0	5.0/10.0	>5.0/>10.0	>5.0/>10.0
Streptomycin sulfate (CK^+^)	0.013/0.025	0.013/0.025	0.013/0.025	0.025/0.05	0.025/0.05

The CE, PE, EE, BE and EO represent the crude extract, petroleum ether extract, ethyl acetate extract, *n*-butanol extract and essential oil of *R. crenulata*, respectively. MIC was the minimum inhibitory concentration. MBC was the minimum bactericidal concentration.

**Table 4:** The MIC and MBC values of the compounds from *R. crenulata* against pathogenic bacteria.

Test Sample	MIC/MBC (mg/mL)				
	*S. dysenteriae*	*S. typhimurium*	*E. coli*	*S. aureus*	*S. albus*
Gallic acid	0.48/>0.48	0.48/>0.48	0.48/>0.48	0.48/0.48	0.48/0.48
Ethyl gallate	0.24/0.48	0.24/0.48	0.24/0.48	0.24/0.48	0.24/0.48
Rosavin	>0.48/>0.48	>0.48/>0.48	>0.48/>0.48	>0.48/>0.48	>0.48/>0.48
Herbacetin	0.24/0.48	0.24/0.48	0.48/>0.48	0.48/>0.48	0.48/>0.48
Streptomycin sulfate (CK^+^)	0.013/0.025	0.013/0.025	0.013/0.025	0.025/0.05	0.025/0.05

**Table 5:** DPPH radical scavenging activity of *Rhodiola* extracts.

Test Sample	Liner Equation	Correlation Coefficient (R)	IC_50_ (µg/mL)
PE	Y = 1.3309X + 1.6760	0.990	314.45 ± 5.15 ^a^
EE	Y = 2.0043X + 2.2291	0.990	24.13 ± 0.50 ^d^
BE	Y = 2.2404X + 1.5111	0.988	36.08 ±0.85 ^c^
WE	Y = 1.3430X + 2.1450	0.997	133.61 ± 3.37 ^b^
CE	Y = 1.7917X + 2.6556	0.986	20.34 ± 0.45 ^e^
EO	Nd	Nd	Nd
BHT(CK^+^)	Y = 1.6905X+2.8796	0.998	17.96 ± 0.82 ^f^

The CE, PE, EE, BE and EO represent the crude extract, petroleum ether extract, ethyl acetate extract, *n*-butanol extract and essential oil, respectively. BHT was the butylated hydroxytoluene. Values represent mean ± standard deviation (*n* = 3). Different letters (i.e., a–f) indicate significant differences among the treatment at *p* = 0.05 level. Nd: not detected, the activity of EO in DPPH radical scavenging test was weak, and its IC_50_ was not detected.

**Table 6:** The IC_50_ values of the four identified compounds from *R. crenulata* in DPPH radical scavenging assay.

Test Sample	Liner Equation	Correlation Coefficient (R)	IC_50_ (µg/mL)
Gallic acid	Y = 2.2023X + 2.2338	0.985	18.03 ± 0.43 ^b^
Ethyl gallate	Y = 1.9158X + 3.6082	0.994	5.33 ± 0.29 ^c^
Rosavin	Y = 2.106X + 3.3835	0.995	5.86 ± 0.27 ^c^
Herbacetin	Y = 2.4227X + 1.7794	0.999	21.35 ± 0.42 ^a^
BHT(CK^+^)	Y = 1.6905X + 2.8796	0.998	17.96 ± 0.82 ^b^

Values represent the mean ± standard deviation (*n* = 3). Different letters (i.e., a–c) indicate significant differences among the treatment at *p* = 0.05 level.
